# Improved targeting of human CD4^+^ T cells by nanobody-modified AAV2 gene therapy vectors

**DOI:** 10.1371/journal.pone.0261269

**Published:** 2021-12-20

**Authors:** Martin V. Hamann, Niklas Beschorner, Xuan-Khang Vu, Ilona Hauber, Ulrike C. Lange, Bjoern Traenkle, Philipp D. Kaiser, Daniel Foth, Carola Schneider, Hildegard Büning, Ulrich Rothbauer, Joachim Hauber

**Affiliations:** 1 Heinrich Pette Institute – Leibniz Institute for Experimental Virology (HPI), Hamburg, Germany; 2 German Center for Infection Research (DZIF), Partner Site Hamburg – Lübeck – Borstel – Riems, Hamburg, Germany; 3 Institute of Experimental Haematology, Hannover Medical School, Hannover, Germany; 4 Department of Anaesthesiology, University Medical Center Hamburg-Eppendorf (UKE), Hamburg, Germany; 5 Natural and Medical Science Institute at the University Tübingen (NMI), Reutlingen, Germany; 6 Pharmaceutical Biotechnology, Eberhard Karls University Tübingen, Reutlingen, Germany; Fudan University, CHINA

## Abstract

Adeno-associated viruses (AAV) are considered non-pathogenic in humans, and thus have been developed into powerful vector platforms for *in vivo* gene therapy. Although the various AAV serotypes display broad tropism, frequently infecting multiple tissues and cell types, vectors for specific and efficient targeting of human CD4^+^ T lymphocytes are largely missing. In fact, a substantial translational bottleneck exists in the field of therapeutic gene transfer that would require *in vivo* delivery into peripheral disease-related lymphocytes for subsequent genome editing. To solve this issue, capsid modification for retargeting AAV tropism, and in turn improving vector potency, is considered a promising strategy. Here, we genetically modified the minor AAV2 capsid proteins, VP1 and VP2, with a set of novel nanobodies with high-affinity for the human CD4 receptor. These novel vector variants demonstrated improved targeting of human CD4^+^ cells, including primary human peripheral blood mononuclear cells (PBMC) and purified human CD4^+^ T lymphocytes. Thus, the technical approach presented here provides a promising strategy for developing specific gene therapy vectors, particularly targeting disease-related peripheral blood CD4^+^ leukocytes.

## Introduction

Adeno-associated virus (AAV)-derived vectors have become a leading gene delivery tool in clinical gene therapies, especially when direct (i.e. *in vivo*) therapeutic gene transfer is needed [1–3]. AAV is a dependoparvovirus, considered to be non-pathogenic in humans. AAV consists of a non-enveloped, icosahedral capsid of ~25 nm and a ~4.7 kb single-stranded DNA genome [4]. The viral genome is flanked by two inverted terminal repeat (ITR) elements, which function as origin of replication and the packaging signal [5, 6].

AAV uses three promoters to produce a family of distinct unspliced and spliced mRNAs encoding various non-structural and structural proteins, which are either involved in viral replication, transcription, integration and virion assembly, or are components of the viral capsid [1, 7]. The substitution of these open reading frames (ORFs) by heterologous transgene cassettes provide space for genetic cargo of up to ~ 4.5 kb. This gutless vector design combined with AAV’s established poor immunogenicity and broad tropism have made AAV-derived constructs the vector of choice in numerous recent clinical gene therapies [2, 8, 9].

The viral capsid comprises the three proteins VP1, VP2 and VP3, which assemble in a ratio of 1:1:10, to form a 60-mer particle. The capsid determines not only the potential of AAV to evade pre-existing immunity, but importantly, also defines viral tropism [10–12]. In primates, thirteen AAV serotypes have so far been identified, differing in the tissues and cell types they infect [13]. AAV serotypes usually possess a rather broad tissue specificity, albeit with preferences for distinct tissues such as the liver in case of AAV8 or AAV9 [14, 15]. However, the inability of all AAV serotypes to specifically, and thus efficiently transduce peripheral blood mononuclear cells (PBMC) represents a considerable technical hurdle, particularly when there is a need for direct *in vivo* gene transfer into human hematolymphoid cells. For instance, the rapidly evolving CAR-T cell therapies would greatly benefit from an *in vivo* approach in activating both CD4^+^ and CD8^+^ T cells towards anti-tumour activity [16, 17]. Another example is infection with HIV, which forms persistent proviruses in CD4^+^ cells. Attempts to excise these proviruses with designer recombinases are progressing steadily, but would also immensely benefit from an *in vivo* CD4-targeted gene delivery platform [18–20].

To overcome this challenge, capsid engineering has recently become a promising strategy to enhance the clinical potential and adapt the tropism of AAV vectors [21, 22]. This strategy either follows a rational design or uses high throughput screening of AAV libraries. The latter either contain variants with point mutations introduced by random mutagenesis of capsid encoding sequences by error-prone PCR, variants with shuffled capsid gene fragments derived from various serotypes and variants, or variants with insertion of random peptides into hypervariable regions of the VP3 capsid protein [2, 21, 23]. Examples for rational design-based approaches encompass genetic fusions of designed ankyrin repeat protein (DARPin)-based targeting ligands fused to the N-terminus of the VP2 capsid protein [24], or insertion of specific heavy-chain-only antibody sequences of camelids (VHH), referred to as nanobodies (Nbs) [25, 26], into a surface loop region common to all capsid proteins [27].

Here, we investigated a set of novel CD4-specific Nbs with the aim of improving AAV vector transduction of human CD4^+^ cells. A set of AAV2 capsid variants were constructed and analysed in cell lines, primary human PBMC and isolated primary human CD4^+^ T lymphocytes. Furthermore, Nb receptor specificity was confirmed in mixed culture experiments. In summary, our data from Nb-mediated targeting of AAV2-derived gene therapy vectors demonstrated increased transduction specificity for human CD4^+^ T cells.

## Materials and methods

### Plasmid construction

The plasmid pAAV2/2 (Addgene #104963) encoding wildtype AAV2 rep/cap genes served as a starting point for capsid optimization and blinding. Amino acid substitutions for capsid optimization (Y444F, T491V, Y500F and Y730F), as well as disruption (blinding) of the primary natural AAV2 heparan sulfate proteoglycan (HSPG) binding site (R585A and R588A) of AAV2 have been described before [21, 28–30]. Genetic modification of the respective nucleotides was conducted by site-directed mutagenesis (SDM) PCR using DNA oligonucleotides in a back-to-back orientation (oligonucleotides oMH19—oMH27; S1 Table) and Q5 DNA polymerase (New England Biolabs, Frankfurt am Main, Germany); PCR programme: initial denaturation 2 min 8°C, 18 cycles denaturation 10” 98°C, annealing 30” 55°C, elongation 6 min 72°C, and final elongation 3 min 72°C. PCR was followed by a “one pot” reaction including a DpnI (Thermo Fisher Scientific, Waltham, MA) digest, 5’ end phosphorylation with T4 Polynucleotide Kinase (New England Biolabs) and T4 DNA ligation (Thermo Fisher Scientific, Waltham, MA). After transformation into *E*. *coli* and plasmid isolation, the introduced nucleotide changes were verified by Sanger sequencing. Sequential rounds of SDM reactions yielded the construct pAAV2/2-o, encoding AAV2 capsid proteins with all four amino acid substitutions affecting proteasomal degradation, as well as pAAV2/2-bo, encoding for capsid proteins which are in addition modified for HSPG binding. AAV2 cap expression yields three capsid proteins, VP1-3: VP1 is translated from unspliced mRNA, whereas VP2 and VP3 are translation products from spliced mRNA. In order to avoid VP2 expression, the major splice acceptor (SA) site was mutated [31, 32]. As described above, SDM PCR (oligonucleotides oMH47 and oMH48; S1 Table) with amplicon ligation and subsequent construct verification was performed, yielding the construct pAAV2/2-bo-SA. In contrast, after mutation of the AAV2 cap start codon, the nascent construct allows only for VP2 and VP3 expression [31, 32]. Analogous to the protocol above, pAAV2/2-bo-sc was generated using oligonucleotides oMH45 and oMH46 (S1 Table).

#### Generation of VP1-modified Nb constructs

Nb and linker sequences were genetically inserted within the GH2/GH3 surface loop region of VP1 [27, 32]. To achieve this a NotI recognition site at the respective location within cap was introduced into pAAV2/2-bo-SA (SDM PCR with oMH67 and oMH68). In parallel to the subsequent ligation reaction, the 5’ linker sequence was introduced by a pair of annealed oligonucleotides (oMH69 and oMH70; S1 Table), yielding pAAV2/2-bo-SA-target-vector. Nb and the 3’ linker sequences were subsequently introduced by Gibson assembly following the manufacturer’s instructions (Gibson Assembly Master Mix, NEB), using the NotI/XcmI linearized pAAV2/2-bo-SA-target-vector fragment and a single PCR fragment comprising the monovalent Nb, 3’ linker and cap sequences were ligated to the cap internal XcmI restriction site (insert fragment). To generate this insert fragment, two single PCR amplicons were fused by overlap extension PCR. First, Nb sequences were PCR amplified from plasmids, using PCR oligos containing 5’ homology to the target vector (forward oligo) as well as the 3’ linker sequence plus 3’ homology to the target vector (reverse oligo) (oligos for each nanobody listed in S1 Table). The second PCR fragment spanning the 3’ linker sequence to the XcmI site within cap was generated using oligos oMH85/86. The resulting VP1-modified Nb constructs (VP1-CD4 -Nb1, -Nb1a, -Nb3, -Nb4 and PepNb) were verified by sequencing. The amino acid sequence of the final constructs is provided in S1 File.

#### Generation of VP2-modified Nb constructs

First, the optimized and blinded VP2 open reading frame was PCR-amplified with DNA oligonucleotides introducing flanking XbaI and BamHI restriction sites from AAV2/2-bo cDNA (oligonucleotides: oMH16 and oMH18; S1 Table). After XbaI/BamHI amplicon digestion, the respective fragment was ligated into the XbaI/BamHI linearized pBC12/CMV vector backbone [33], resulting in construct pBC12-CMVint-VP2. Next, Nb sequences were genetically fused via a linker sequence to the 5’ end of the VP2 open reading frame to achieve expression of amino-terminal Nb-linker-VP2 fusion peptides. To this end, a three fragment ligation strategy was applied comprising the HindIII/XbaI linearized pBC12-CMVint-VP2 vector fragment, the linker fragment consisting of the annealed oligonucleotides oMH33 and oMH34 with XbaI/XhoI-compatible ends, and the Nb PCR insert fragment with XhoI/HindIII compatible ends (Nb-specific oligos used for PCR amplification are listed in S1 Table). All final constructs (VP2-CD4-Nb1, -Nb1a, -Nb3, -Nb4, -Nb5 and PepNb) were verified by Sanger sequencing. The amino acid sequences of the final constructs are provided in S1 File.

#### Generation of VP1-modified bivalent Nb constructs

Tandem VP1-biCD4-Nb1 and control VP1-biPepNB constructs were designed as the sequence of two full-length monomeric Nbs fused by a linker sequence and were generated from synthesized DNA fragments (GeneART-Thermo Fisher Scientific, Waltham, MA) containing NotI/XcmI restriction sites, linker sequences flanking each Nb and VP1 sequences with optimized and blind modifications. Both GeneArt synthesis plasmids and pAAV2/2-bo-SA-target vector were digested with NotI/XcmI and the bivalent Nb insert was ligated into the VP1-vector, resulting in pAAV2/-bo-SA-VP1-biCD4 and -biPepNB. Final constructs were verified by DNA sequencing. The amino acid sequence is provided in S1 File.

### Cell culture

All cell lines used in this study were described before and cultivation was performed under standard conditions in humidified atmosphere at 37°C and 5% CO_2_. Adherent cell lines HEK293T (ATCC: CRL-3216), HeLa (ATCC: CCL-2) and HeLa TZMbl (NIBSC: ARP5011) were passaged using 0.05% trypsin-EDTA (Pan-Biotech, Aidenbach, Germany) and cultured in DMEM growth medium (Pan-Biotech, Aidenbach, Germany) supplemented with 10% fetal calf serum (Biochrom, Berlin, Germany), 2 mM L-glutamine and 50 U/mL penicillin/streptomycin antibiotics (Biochrom, Berlin, Germany). Suspension T cell lines SupT1 (ATCC: CRL-1942) and Jurkat 1G5 (NIBSC: 5010) were cultivated in RPMI 1640 growth medium (Biozyme Scientific) supplemented with 10% fetal calf serum (Pan-Biotec, Aidenbach, Germany) and 50 U/mL penicillin-streptomycin.

PBMC were isolated from buffy coats of healthy donors. Isolation of CD4^+^ T cells from buffy coats was carried out using the RoboSep negative selection human CD4^+^ T cell enrichment kit in conjunction with a RoboSep automated cell separator (Stemcell Technologies, Köln, Germany) according to the manufacturer’s instructions. Isolated cells were cultivated in RPMI 1640 (Biozym Scientific, Hessisch Oldendorf, Germany) supplemented with 10% FCS, 100 U/mL penicillin-streptomycin, 2 mM L-glutamine and 500 U/mL recombinant human IL-2 (Sigma-Aldrich, Hamburg, Germany).

Experimental work with primary human cells was reviewed and approved by the relevant German authority, the local ethics commission (Ärztekammer Hamburg; PV4666 and WF-010/2011). The respective specimen/data were analysed anonymously.

### AAV particle production, purification and quantification

AAV production in HEK293T cells via polyethylenimine (PEI; Sigma-Aldrich, Hamburg, Germany) transfection was performed as described before [34, 35]. Briefly, 1 day prior transfection 4x10^6^ cells were seeded per 10 cm dish. Transfection was conducted with a 4:1 PEI:DNA ratio in Opti-MEM (Thermo Fisher Scientific, Waltham, MA) with a total of 10.5 μg plasmid DNA per dish (i.e. 42 μg PEI:10.5 μg DNA). The PEI transfection protocol was conducted following the manufacturer’s instructions in a final volume of 6 mL per dish. In all co-transfection reactions, the helper plasmid pAdDeltaF6 (Addgene #112867) and the AAV2 vector genome pscAAV-CAG-GFP (Addgene #83279) encoding the GFP transgene under a constitutive CAG promoter were included. Further plasmids were added to the mix as required (opt: pAAV2/2-o; opt-blind: pAAV2/2-bo; VP1-Nb: pAAV2/2-bo-sc + pAAV2/2-bo-SA-VP1-NbX; VP2-Nb: pAAV2/2-bo + pBC12-CMVint-VP2-NbX). All plasmids were co-transfected in equimolar quantities. Thirty hours post transfection, 6 mL of fresh medium was added to the cultures. Three days post transfection, cells were scraped off the dish and combined with the supernatant. After centrifugation (15 min, 2,800 x g), supernatant was discarded, the cell pellet resuspended in 800 μL lysis buffer (150 mM NaCl, 50 mM Tris-HCl pH 8.0 in water) and transferred into a 1.5 mL reaction tube. Four cycles of freeze/thaw (-80°C for 20 min and 37°C for 5 min) released AAV particles from producer cells. Subsequent benzonase treatment (50 U, 37°C, 1 h; Sigma-Aldrich) reduced residual plasmid DNA and RNA contamination. After separation from cell debris (20,000 x g, 4°C, 15 min), aliquots of the resulting crude AAV production was stored at -80°C until use.

For indicated experiments (i.e. CD4^+^ T cells and PBMC transduction), iodixanol step gradient purification of crude AAV production was conducted. After large scale production (twenty 10 cm dishes, 9 mL lysis buffer, 450 U benzonase), 9 mL of crude AAV preparation was purified over an iodixanol step gradient as described before [34]. After ultracentrifugation (4°C, 350,000 x g, 90 min; Beckman 70 Ti rotor) the 40% iodixanol fraction containing the AAV2 particles was collected. Washing, PBS-Pluronic F68 buffer (Gibco) exchange and further concentration was achieved by filter column centrifugation (Amicon Ultra-15, 100 kDa MWCO; Sigma-Aldrich, Hamburg, Germany) as described before (https://www.addgene.org/protocols/aav-purification-iodixanol-gradient-ultracentrifugation/). Aliquots of purified AAV vector preparations were stored at -80°C until use.

Vector titers were determined by qPCR. For this, 1 μL of the AAV vector preparation was subjected to DNaseI and a subsequent Proteinase K treatment, to eliminate residual plasmid DNA contamination and release viral vector genomes from the capsids. A 100 μL reaction containing 1 μL AAV vector preparation (10 U DNaseI in 1x DNaseI buffer; New England Biolabs, Frankfurt am Main, Germany) was incubated at 37°C for 16 h followed by DNaseI inactivation at 75°C for 30 min. Next, 2 μL of Proteinase K (20mg/mL; QIAGEN, Hilden, Germany) was added and incubated at 55°C for 2 h. Proteinase K was inactivated at 95°C for 30 min. From this reaction, 1 μL was subjected to absolute quantification of vector genomes using a plasmid standard as reference (pscAAV-CAG-GFP). The Platinum^®^ SYBR^®^ Green qPCR SuperMix-UDG kit (Thermo Fisher Scientific, Waltham, MA) was used according to the manufacturers’ instructions in combination with an Applied Biosystem 7500 real-time PCR cycler (qPCR programme: initial denaturation 95°C 3 min; 40 cycles of denaturation 95°C 15” and annealing/elongation 60°C 30”, and a subsequent melting curve; oligonucleotides oMH93 and oMH94 are listed in S1 Table). Capsid quantification from iodixanol step gradient-purified vector preparations were conducted using an AAV2 titration ELISA kit (PROGEN, Heidelberg, Germany) following manufacturer’s instructions.

### Immuno-gold electron microscopy

Iodixanol step gradient purified AAV2 samples were adsorbed to glow discharged carbon coated grids for 10 min. After incubation with blocking solution for protein A/G gold conjugates (AURION, Wageningen, The Netherlands) for 5 min, samples were incubated with the primary antibody (10 μg/mL, polyclonal goat anti-alpaca IgG; Jackson ImmunoResearch, West Grove, PA) for 30 min followed by 5 min washing. Subsequently, the secondary antibody was added for 15 min (1:10, rabbit anti-goat IgG 10 nm gold-conjugated; Sigma-Aldrich, Hamburg, Germany). After washing with ddH_2_O, samples were stained with 1% uranyl acetate and air dried. Transmission electron micrographs were taken with a FEI Tecnai G20 equipped with an Olympus Veleta CCD camera at 80 kV. Representative images with corresponding scale bars were cropped for clarity.

### Western blot

Capsid composition was analysed by Western Blot. Iodixanol-purified viral vector particles were concentrated using the StrataClean Resin (Agilent Technologies, Ratingen, Germany) according to manufacturer’s instructions, separated on a 8% SDS-PAGE gel (5x10^10^ capsids per lane) and subjected to standard Western blotting procedure using the following primary antibodies: hybridoma cell supernatant containing AAV2 specific antibody A69 (recognizing VP1/VP2 proteins [36], 1:10 dilution) (PROGEN, Heidelberg, Germany). Anti-mouse IgG HRP-conjugated antibody was used as secondary antibody (Cayman Chemical—Biomol, Hamburg, Germany; 1:2,000 dilution) and signals were detected using the SuperSignal^™^ West Pico Chemiluminescent Substrate or SuperSignal^™^ West Femto Maximum Sensitivity (ThermoFisher Scientific, Waltham, MA) in combination with a Fusion FX Vilber Lourmat system (Peqlab).

### Capsid thermal stability assay

Capsid thermal stability assays were performed as described before [37]. Briefly, per reaction 1x10^10^ viral capsids of a vector preparation were diluted in PBS, incubated at indicated temperatures for 15 min and then further diluted in PBS. The temperature gradient (65 to 85°C) was generated by a LightCycler^®^ 96 System (Roche Life Science) with the following program: 2 cycles (10 sec at 37°C; 900 sec at temperature gradient; 10 sec at 37°C), 1 cycle (30 sec at 37°C). Samples were transferred to a nitrocellulose membrane using a vacuum blotter, blocked (5% milk in TBS-T) and incubated with hybridoma cell supernatant containing anti-AAV2 specific antibody A20 (recognizing intact capsids) (PROGEN, 1:5 dilution). Anti-mouse IgG HRP-conjugated antibody was used as secondary antibody (Cayman Chemical—Biomol, Hamburg, Germany; 1:2,000 dilution) and signals were detected using SuperSignal^™^ West Pico Chemiluminescent Substrate or SuperSignal^™^ West Femto Maximum Sensitivity (ThermoFisher Scientific, Waltham, MA) in combination with a Fusion FX Vilber Lourmat system (Peqlab).

### AAV transduction and flow cytometry

If not further specified, cells were transduced with crude AAV2 lysates at the indicated genome copies per cell (gc/cell). One day prior to infection, 2.5x10^4^ cells were seeded per well in a 12-well dish. In mixed culture experiments, HeLa wildtype and HeLa TZMbl cells were seeded in an approximate 1:1 ratio with a total of 2.5x10^4^ cells per well 24 h prior to transduction. For transduction of primary CD4^+^ T cells and PBMC from buffy coats, cultures were pre-stimulated with CD3/CD28 magnetic beads (Thermo Fisher Scientic, Waltham, MA) for 24 h according to the manufacturer’s instructions. After pre-stimulation, cells were counted and seeded in a 96-well plate with 4x10^4^ cells per well. Iodixanol step gradient-purified AAV particles were added to the cultures at the indicated genome copies per cell in the presence of 500 U/mL recombinant human IL-2. In all cell culture experiments, cells were harvested three days post transduction, washed with PBS and stained with PE-Cy7 conjugated anti-CD4 antibody (RPA-T4; eBioscience—Thermo Fisher Scientic, Waltham, MA) in PBS with 2% FCS and 1 mM EDTA at 4°C for 30 min. After two rounds of washing with PBS, cells were analysed for CD4 and/or GFP expression. Data was acquired on a Canto II (BD Biosciences, Heidelberg, Germany) or LSR Fortessa (BD Biosciences, Heidelberg, Germany) device.

### AAV binding and internalization

Capsid binding and internalization assays were essentially conducted as described before [38]. In brief, HeLa wt or TZMbl cells were seeded at a density of 2.5x10^5^ cells per 12well 24h prior to infection. Thirty minutes prior to transduction, cells were cooled to 4°C and subsequently inoculated with selected purified AAV2 preparations at 10.000 gc/cell. After 1h incubation at 4°C, cells were washed four times with ice-cold PBS before harvesting cells with a cell scraper (AAV2 binding samples). For analysis of AAV2 internalization, cells with bound AAV2 particles were transferred to 37°C and incubated for an additional hour. Afterwards cells were harvested with trypsin and washed three times with PBS (AAV2 internalization samples). Genomic DNA was isolated using the QIAamp DNA Blood kit (QIAGEN) following manufacturer’s instructions. Quantification PCR of vector genomes was carried out as described above using oligonucleotides oMH93 and oMH94 (S1 Table). Quantification of cellular genomes was performed using a Taqman qPCR specific for the human beta-globin gene (HBG) in combination with Platinum Quantitative PCR SuperMix-UDG (Live Technologies) following manufacturers instructions (oligonucleotides HBG-F, HBG-R and HBG-probe listed in S1 Table).

### Data analysis and statistics

All flow cytometry data was analysed with FlowJo v10.7 software (Tree Star, Ashland, OR) and processed in GraphPad v9 (Prism, Irvine, CA). Data represent means with error bars showing standard deviation (number of experiments indicated in figure legends). P values were calculated to evaluate statistical significance (GraphPad v9) and indicated in the figures by asterisks: * p < .05, ** p < .01, *** p < .001, **** p < .0001. P values in Figs 3 and 4 were calculated using unpaired t-test with Welch correction and FDR 0.01. P values in Fig 5 and S1 Fig was calculated by ordinary one-way ANOVA. P values in S2 Fig were calculated using paired t-test and FDR 0.01.

## Results

### Generation of nanobody-engineered AAV2 particles

**Fig 1:**
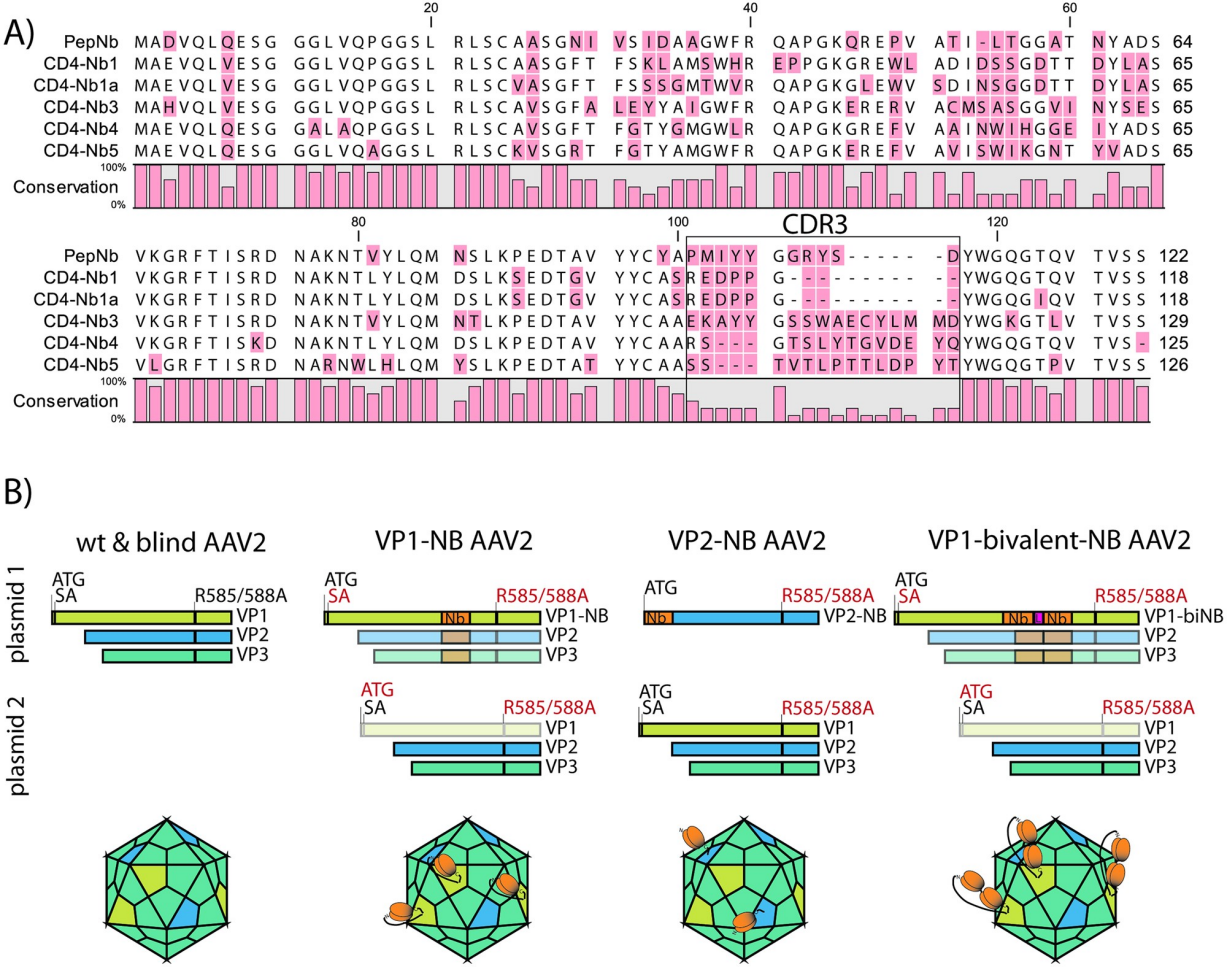
Nanobody (Nb) sequences and AAV2-Nb design. (A) Amino acid alignment of the Nb sequences used in this study. Nb annotation is in accordance with the study by Traenkle and coworkers [39], which describes thorough Nb characterization and application for *in vivo* imaging. Of note, CD4-Nb1a is not specified in Traenkle et al., as its complementary determining region 3 (CDR3, black box) is identical to CD4-Nb1 (amino acids 101–117 in the alignment). (B) Plasmid constructs used for production of indicated AAV2 particles and schematic representation of the resulting capsids. Equimolar amounts of plasmids were co-transfected into HEK293T cells (see Methods). For clarity, adenoviral helper plasmid and vector encoding the eGFP reporter included in all co-transfections are not depicted. Essential changes to optimized viral capsid proteins VP1-3 are indicated: red = mutated / black = wt. Disabled open reading frames are greyed out. ATG = start codon; SA = splice acceptor site; Nb = full length nanobody sequence as presented in A (orange); L = glycine-serine linker genetically fusing nanobody monomers in the bivalent constructs (pink); R585A/R588A = Arg to Ala amino acid substitution at residues 585 and 588 (blind). AAV capsids were rendered with help of the browser tool by Antonio Negrón [42].

Recently, a set of five novel Nbs (amino acid sequence depicted in Fig 1A) have been developed that specifically recognize the human CD4 receptor in its native state with high affinity [39]. Here, we assayed these Nbs in the context of AAV aiming for AAV capsid variants with tropism for CD4^+^ T lymphocytes. We therefore synthesized the cDNAs encoding the respective Nbs and used them for genetic modification of AAV2 capsid (*cap*) genes. For capsid modification purposes, large heterologous sequences are either inserted into the GH2/GH3 surface loop of the VP3 common region of the VP1 proteins [27], or fused to the amino-terminus of VP2 [40]. Following both strategies, selected CD4 Nb-encoding sequences, including Nb tandem constructs, were fused in frame with VP1 and/or VP2 coding sequences, resulting in AAV2 capsid particles displaying human CD4-specific Nbs on their surface (Fig 1B). To control for CD4-nanobody specific binding, we additionally included a nanobody (PepNb), which binds a non-related peptide epitope [41].

In addition, to optimize vector transduction efficiency, four surface-exposed capsid residues were mutated (Y444F, T491V, Y500F, and Y730F) in order to prevent their phosphorylation, a post-translational modification that otherwise may lead to ubiquitination, followed by proteasomal degradation of incoming vector in the target cell’s cytoplasm [21]. In addition, AAV2 *cap* R585A and R588A were modified to prevent binding to heparin sulphate proteoglycan, the primary receptor of AAV2 (blinding) [21]. All vector Nb-modifications were carried out in this modified capsid backbone. Vectors were packaged with self-complementary vector genomes [43] encoding a eGFP marker gene driven by a constitutive CAG promoter [43]. Of note, our production strategy for VP2-Nb modified vectors results in the co-expression of wt and Nb-modified VP2 proteins in the producer cells (Fig 1B). Hence, hybrid-capsids containing both wt and Nb-modified VP2 are likely present in all our VP2-Nb vector preparations.

**Fig 2:**
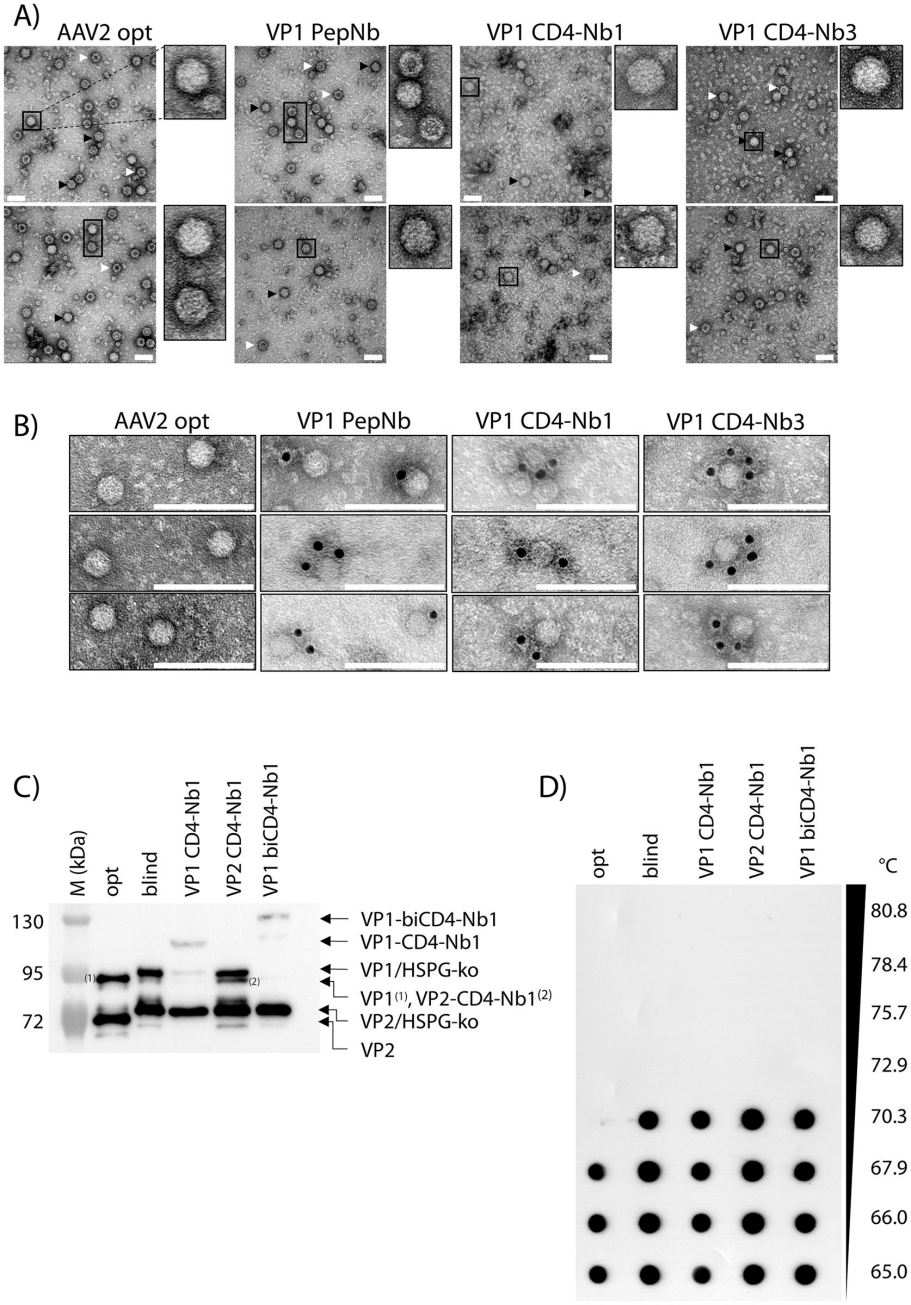
Electron microscopic analysis of Nb-containing AAV2 capsids. (A) Purified AAV2 particles were subjected to negative staining electron microscopy. Representative images show vector genome-containing (black arrow) and empty (white arrow) capsids. Individual capsids are magnified. Scale bar 50 nm. (B) Immuno-gold staining of purified AAV2 particles indicate accessible Nbs at the capsid surface. Images were cropped to show representative capsids. Scale bar 100 nm. (C) Western blot of purified vector particles using a VP1/VP2-specific antibody (A69). Individual bands are labeled; HSPG-ko indicate shifted protein bands compared to opt AAV2 due to R585/588A heparan sulfate proteoglycan (HSPG) blinding. (D) Capsid thermal stability assay of purified vector particles using AAV-specific antibody A20 detecting intact capsids. Temperature gradient is indicated.

Vectors were produced by transient co-transfection of HEK293T cells (see Materials and methods). Selected gradient-purified vector batches were analysed by electron microscopy and showed a wildtype-like phenotypic AAV appearance, regardless of whether the viral particles were Nb-modified (Fig 2A). Furthermore, immune-gold staining using a primary antibody against alpaca IgG confirmed accessibility of Nb moieties on the capsid surfaces of Nb-modified vector preparations (Fig 2B). Western blot analysis confirmed incorporation of nanobodies as indicated by the VP1/VP2 protein bands being upshifted by approximately 15 kDa per nanobody moiety (Fig 2C). Using qPCR and capsid ELISA determining genomic and capsid titer revealed that the packaging efficiency, i.e. the ratio of capsids/genomes, are very similar for control vectors (opt: 11.13 and blind: 6.91, respectively) and Nb-modified vectors (VP1-CD4-Nb1: 7.17, VP2-CD4-Nb1: 7.88 and VP1-biCD4-Nb1: 8.74, respectively), indicating unaffected genome packaging in Nb-modified particles (S2 Table). Furthermore, capsid thermal stability was assayed by gradually heating vector capsids and specific detection of intact capsids [37]. AAV blind and Nb-modified vector capsids show identical thermal stabilities (up to 70.3°C), indicating that Nb-engineering does not negatively affect capsid stability (Fig 2D).

In summary, these data show that our Nb-modified AAV2 particles have AAV2 wildtype-like morphology and genome packaging capacity and display the Nbs on their outer capsid surface.

### Transduction of CD4 expressing cell lines

**Fig 3:**
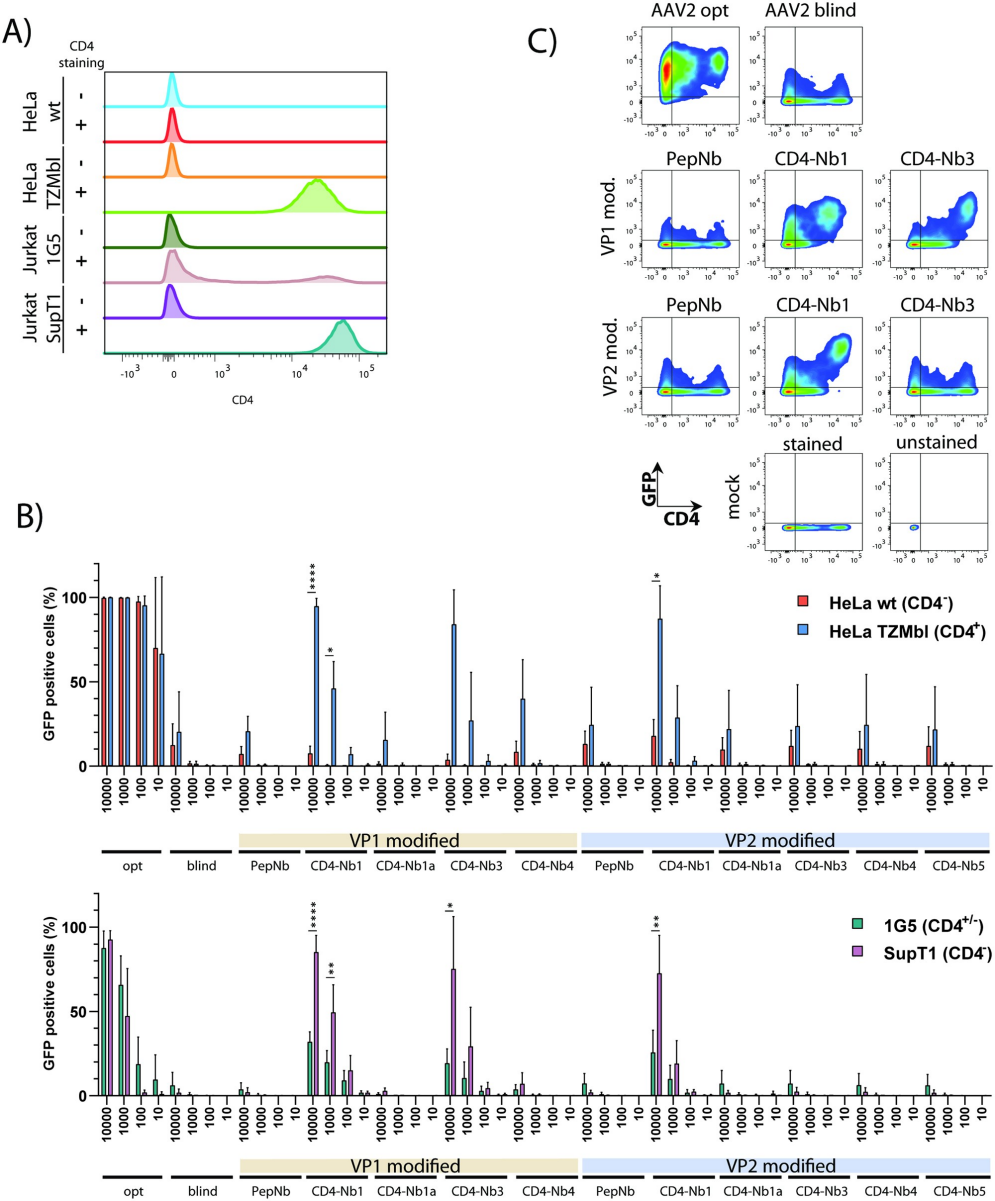
AAV2 CD4-Nb-containing particles efficiently transduce CD4 expressing cells. (A) CD4 expression on cell lines used in this study. Cells were stained with anti-CD4-Cy7 antibody and compared to unstained mock controls. The CD4 signal in mock cells was manually adjusted to 100 arbitrary units and compared with stained cells by flow cytometry. Histograms were normalized by modal mode to visualize comparable peak heights. (B) Two HeLa (wt and TZMbl) and two T lymphoid (1G5 and SupT1) cell lines were transduced with the indicated viral vector genome copies per cell (gc/cell). Three days post infection the % of eGFP positive cells was determined by flow cytometry; n = 2–4. Error bars indicate the SD. (C) 1G5 cells were transduced with the indicated AAV2 particles at a concentration of 10,000 gc/cell. Three days post transduction cells were stained for CD4 expression and analyzed for GFP expression by flow cytometry. Representative data are shown.

First, various cell lines were analysed with respect to CD4 surface expression by flow cytometry. As shown, CD4-deficient HeLa wildtype (wt) and CD4^+^ HeLa TZMbl cells displayed the expected phenotypes, lacking or expressing the CD4 molecule on the cell surface (Fig 3A). Human Jurkat 1G5 T cells expressed CD4 moderately, while the related SupT1 cells were characterized by strong CD4 expression (Fig 3A). Next, these cell lines were transduced individually with AAV vector preparations, comprising the controls AAV2opt (Y444F, T491V, Y500F, Y730F) and AAV2blind (Y444F, T491V, Y500F, Y730F, plus R585A, R588A), as well as a set of capsid (Nb)-engineered vectors, including the PepNb as negative Nb control. Transduction of HeLa cells with the control vector AAV2opt resulted in high transduction rates over the entire range of input vector genome copies per cell (gc/cell), ranging in every experiment from 10,000 to 10 gc/cell (Fig 3B, upper panel). These results were observed irrespective of whether transduced HeLa cells were negative (HeLa wt) or positive for the CD4 receptor (HeLa TZMbl).

Analysis of the control vectors AAV2blind and PepNb revealed comparably low transduction rates only at the highest concentration of 10,000 gc/cell, confirming a strong reduction in infectivity due to elimination of primary receptor binding capability. In contrast, inspection of the various CD4 Nb-specific vectors (CD4-Nb), particularly in the case of CD4-Nb1 and CD4-Nb3 (schematically outlined in Fig 1A), demonstrated maximal transduction in a CD4 receptor-dependent manner at high vector genome copies (Fig 3B, upper panel). Interestingly, CD4-Nb1 displayed a similar transduction efficiency independently of the site of Nb sequence insertion (internal loop in VP1 versus amino-terminus in VP2), while in contrast, CD4-Nb3 only performed well when VP1 was genetically modified. Similar results were obtained in the T cell lines SupT1 and Jurkat 1G5 (Fig 3B, lower panel). Although transduction efficiencies were overall lower across the entire range of input vector (i.e. gc/cell), CD4-Nb1 and CD4-Nb3 demonstrated comparable and maximal transduction rates of SupT1 cells in particular, which stained highly positive for CD4 (Fig 3A). As seen above, the CD4-Nb3 vectors only positively affected transduction of CD4^+^ cells when displayed at the highest protrusion as part of VP1. These data were also confirmed using Jurkat 1G5 cells transduced with the CD4-Nb1 or CD4-Nb3 vectors (10,000 gc/cell) at day 3 post transduction (Fig 3C). Inspection of the flow cytometry plots revealed enhanced transduction of cells strongly expressing CD4. As above, this effect was observed for CD4-Nb1 VP1- and VP2-modified viral particles, as well as for CD4-Nb3 VP1-modified AAV2.

To gain a more comprehensive understanding on the cell transduction process, we investigated vector entry and trafficking of Nb-modified capsids. We used the top candidate VP1-modified CD4-Nb1 and–Nb3 vectors and compared these to the VP1-PepNB negative control and the AAV2 opt vector as positive control on HeLa wt (CD4 negative) and HeLa TZMbl (CD4 positive) cells. Our results show that both CD4-Nb vectors do bind (or rather attach) to HeLa wt and TZMbl cells to a comparable extent as AAV2 opt vector, irrespective of CD4 cell surface expression (S1 Fig, left panel). In contrast, PepNb vector particles bind/attach significantly less to both cell lines compared to the AAV2 opt vector (S1 Fig, left panel). A differential pattern emerged when analysing particle internalization. In HeLa wt cells, clearly all Nb-modified capsids are significantly less internalized compared to AAV2 opt vector particles, consistent with the mutated HSPG-binding motif and the lack of CD4 expression on the surface of these cells (S1 Fig, right panel). In HeLa TZMbl cells CD4-Nb1 capsids, the best performing construct in previous experiments, was internalized over 4fold more effectively compared to AAV2 opt vectors in line with our flow cytometry data (Fig 3B). CD4-Nb3 capsids, however, were internalized less efficiently compared to AAV2 opt vectors in our assay (S1 Fig, right panel). The reduced internalization of CD4-Nb3 compared to CD4-Nb1 is also reflected by a lower GFP transgene expression in our flow cytometry data (Fig 3B). Whether this is a matter of timing and CD4-Nb3 is internalized with different kinetics remains to be tested.

Taken together, these experiments indicated that CD4-Nb1 and CD4-Nb3 are useful reagents for targeting AAV2-derived gene vectors to cells, particularly those expressing CD4 on the surface.

### Preferential targeting of CD4^+^ cells in mixed cell cultures

**Fig 4:**
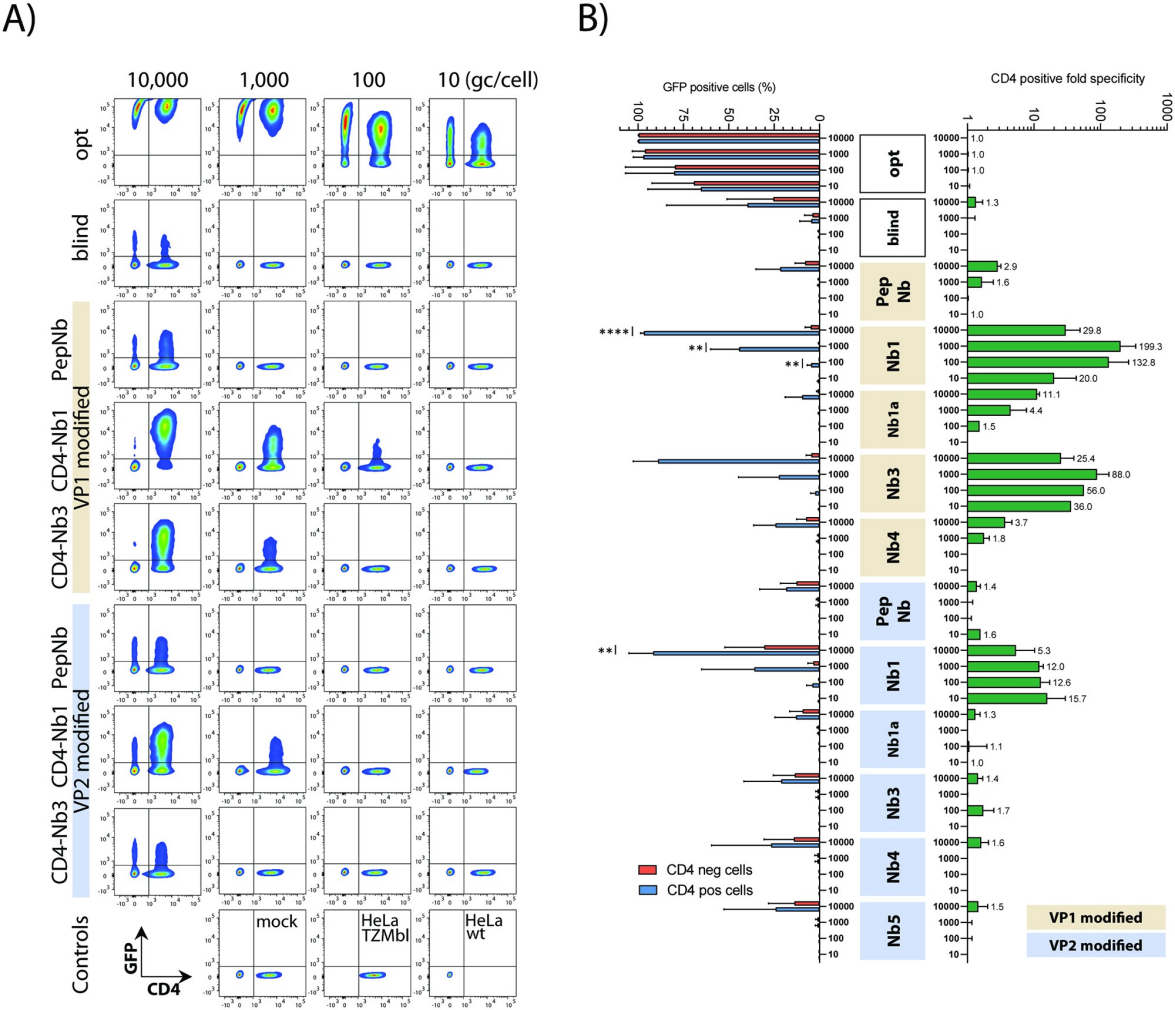
AAV2-CD4 Nb specificity in mixed culture experiments. (A) Representative analysis of a mixed culture experiment. HeLa wt (CD4 negative) were mixed with HeLa TZMbl (CD4 positive) in a ratio of 1:1 prior to AAV2 transduction and subsequently transduced with different virus dilutions. Three days post transduction, cells were harvested, stained for CD4 and analyzed for GFP expression by flow cytometry. (B) Cumulative data from independent HeLa mixed culture experiments. Indicated are the relative frequencies of GFP positive cells for CD4 positive and negative cells (on the left). AAV2 CD4-specific transduction is calculated as ratio from the individual cell populations (fold CD4 positive over CD4 negative). Fold changes are indicated (on the right); n = 2–6, presented are means with SD, significant differences indicated with asterisks: * p < .05, ** p < .01, *** p < .001, **** p < .0001.

Next, we evaluated CD4-specific cell targeting by AAV2 CD4-VP1 and CD4-VP2 vectors in competition experiments, where two distinct cell populations, CD4-negative HeLa wt cells and CD4-positive HeLa TZMbl cells, were mixed in a ratio of 1:1. In agreement with the results above, addition of the respective CD4-Nb-modified vector particles and control vectors to these mixed cell cultures, followed by flow cytometry, clearly revealed preferred and highly significant transduction of the CD4-expressing subpopulation in the respective mixed cultures (Fig 4A and 4B). In contrast, the AAV2opt vector again efficiently and promiscuously transduced target cells in a dose dependent manner and regardless of whether the CD4 surface receptor was expressed (compare Figs 3B and 4A). The remaining vector controls, AAV2blind and PepNb, also showed indiscriminate cell transduction, however, as recorded before only at the highest vector particle concentration used (10,000 gc/cell) (Fig 3B).

Subsequently, the relative frequencies of eGFP-specific signals in CD4^+^ and CD4^-^ cells from multiple independent experiments were calculated and presented as CD4^+^/CD4^-^ ratios (Fig 4B). These data confirmed the expected CD4-Nb-dependent increase in transduction efficiencies, especially when the Nb1- and Nb3-encoding sequences were displayed as part of VP1, and moderate or low input vector particles (i.e. 1,000 to 10 gc/cell) were used. The respective frequencies indicated a 20- to 199-fold increase in the transduction rate of CD4^+^ over CD4^-^ tissue culture cells (Fig 4B).

It has been previously reported that under certain conditions homologous Nb tandem-repeats may increase the affinity of a specific Nb [44]. Therefore, two copies of CD4-Nb1 connected by a long flexible glycine serine linker were genetically inserted into VP1 as a homodimer (depicted in Fig 1B and S1 File) and tested as described above. As control for Nb specificity, two copies of the Pep-Nb were cloned into VP1 in parallel. The analyses revealed that bivalent CD4-Nb1 (i.e. a tandem Nb1 repeat), although displaying significant specificity comparable to the monovalent format, is not more potent in transducing CD4^+^ cells (S2 Fig). This may be explained by some structural interference, since construction of the bivalent CD4-Nb1 vector required an intragenic insertion of 885 nucleotides (nt), a size that is clearly larger than the maximal heterologous insertion reported for this site before, which comprised a 720 nt sequence encoding the mCherry marker protein [32].

In summary, these data indicate that CD4-Nb-modified AAV2 vectors allow specific targeting of CD4 receptor-expressing cells in mixed cell cultures. Thereby, Nb incorporation in VP1 shows a higher specificity compared to VP2-nanobody variants and CD4-Nb1 as well as CD4-Nb3 are the most potent candidates of our Nb panel. Moreover, monovalent Nb1 capsids are superior compared to bivalent Nb1 capsids, even at relatively low vector doses.

### Enhanced transduction of primary human CD4^+^ cells

**Fig 5:**
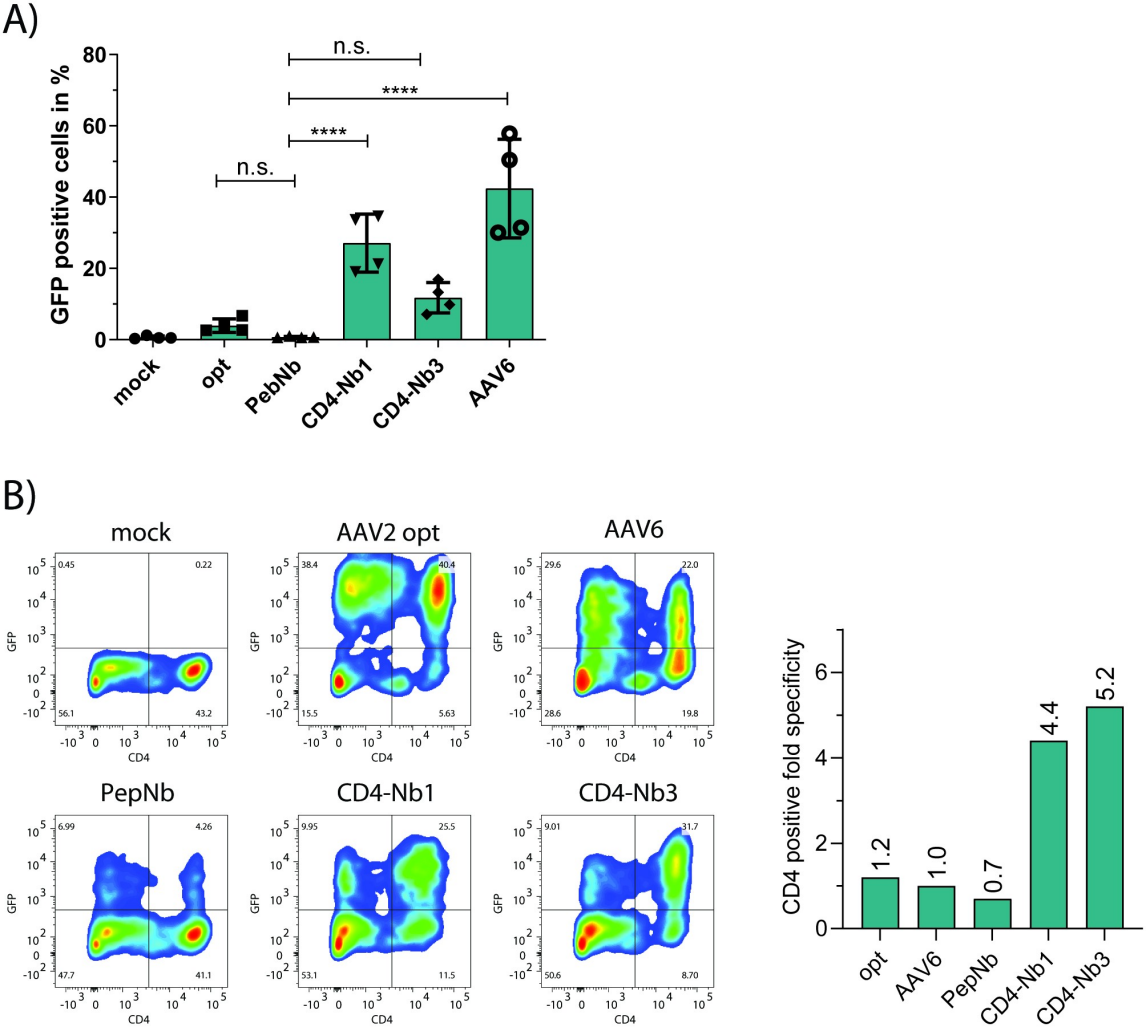
AAV2 VP1 CD4 Nb specificity in primary CD4^+^ T and PBMC. (A) Primary human CD4^+^ T cells from two healthy donors were CD3/CD28 activated and transduced with the indicated vector stocks (20,000 gc/cell). Three days post transduction, cells were stained for CD4 and analyzed for GFP expression by flow cytometry. Error bars indicate the SD; n = 4; n.s. not significant; **** p < .0001. (B) Primary human PBMC were CD3/CD28 stimulated and subsequently infected with the indicated vector stocks (10,000 gc/cell). Three days post transduction, cells were stained for CD4, and GFP expression was assessed via flow cytometry (left panels). Fold specificity for CD4 positive cells (right panel) was plotted from relative eGFP positive populations in CD4 negative and positive cells. Data from one representative experiment.

Next, we assayed our novel vectors in a clinically more relevant setting, i.e. regarding their ability to transduce primary human CD4^+^ T cells as well as human PBMC. First, purified CD4^+^ T cells from two healthy donors were treated with indicated targeting vectors. As controls, we included a mock transduction control, AAV2opt and PepNb, as well as an AAV6 vectors. We included the latter because of reports about AAV6’s ability to transduce various human primary cells, including dendritic cells (DC), CD34^+^ hematopoietic stem and progenitor cells (HSC), and CD8^+^ and CD4^+^ T cells [45–47]. As opposed to the human cell lines investigated above, the transduction efficiencies were clearly lower in primary cells (Fig 5A). Nonetheless, we observed successful transduction of CD4^+^ T lymphocytes, particularly by AAV2 CD4-Nb1 and AAV6. To elucidate transduction specificities, we then transduced a human PBMC culture and calculated ratios of transduced CD4^+^/CD4^-^ cells as outlined above. AAV2 CD4-Nb1- and Nb3-modified vector particles transduced CD4^+^ cells about 4- to 5-fold more efficiently than cells lacking or only weakly expressing the CD4 receptor (Fig 5B). As expected, due to its rather broad tropism, AAV6 failed to specifically transduce CD4^+^ PBMC in this analysis.

Taken together, these data demonstrate that CD4 Nb capsid-engineered AAV2 vectors show an improved tropism for CD4^+^ human PBMC.

## Discussion

Gene therapy vectors based on AAV have become one of the leading delivery tools in human clinical gene therapy trials [2, 8, 9, 14]. The AAV serotypes known to date transduce numerous tissues and cell types [13]. However, to expand the application of AAV gene therapy vectors further, improved and more specific targeting of distinct cell types is needed. For example, therapeutic gene transfer into human disease-associated leukocytes would greatly benefit from the availability of such novel AAV vector constructs. Here, we successfully altered AAV vector tropism towards CD4 and thereby its clinical applicability by capsid engineering using a rational-design based approach.

Previously, AAV capsid modification to preferentially target CD4^+^ human T cells made use of engineered VP2 capsid proteins that were equipped with high-affinity CD4 receptor binding designed ankyrin repeat proteins (DARPin) [40]. Indeed, human CD4^+^ cell lines and primary human CD4^+^ lymphocytes, including CD4^+^ cells present in the spleen of a humanized mouse model, were successfully transduced with such DARPin-engineered AAV2 vectors [40]. However, more recent studies in rhesus macaques demonstrated that CD4-specific DARPin-modified AAV6 vectors had a biodistribution comparable to unmodified AAV6 *in vivo* [48]. This finding may be related to the fact that the respective animals were infused with a mixture of DARPin-modified and unmodified AAV6 vectors [48].

In principle, receptor-specific antibodies, for example single-chain antibodies, would present an alternative approach to genetically modify AAV capsids. Unfortunately, however, the assembly of nascent AAV particles in the cell nucleus with its reducing conditions is detrimental to proper antibody folding, thereby hampering this approach [21]. A strategy circumventing this difficulty and at the same time exploring the high selectivity and specificity of antibodies is the use Nbs. Nbs are defined as antigen-binding domains of camelid heavy-chain-only (VHH) antibodies [25]. Due to their high homology to human IgVH3 molecules, Nbs are known to be weakly immunogenic in humans and are characterized by their small size, antibody-like affinities and specificities, high stability and good solubility [26]. Therefore, Nbs are increasingly explored in biotechnology and medicine, particularly as tools for diagnosis and imaging [49].

In the present study, we chose newly generated CD4-specific Nbs to modify AAV2 capsids. We employed the alpaca derived Nbs CD4-Nb1 and CD4-Nb3, which were previously reported to display affinities (i.e. K_D_ values) for human CD4 of ~5 nM and ~75 nM, respectively [39]. Interestingly, the presentation of these Nbs on the surface of the capsid as part of VP1 protein appeared to be more effective than their display as fusion to the amino-terminus VP2 (compare Figs 3 and 4). This might be partially explained by our VP2-Nb vector plasmid combination, which allows for co-expression of wt and Nb-modified VP2 proteins in the producer cells and therefore generates likely hybrid-capsids containing both forms of VP2 proteins. On the other hand, insertion of a heterologous sequence, including Nb-encoding sequences, at the same position in VP1 has been successfully pioneered before demonstrating its effectiveness [27, 32]. Likewise, the identical insertion of a homologous tandem repeat of CD4-Nb1 into VP1 also resulted in enhanced, but not further improved, specificity of this vector for CD4^+^ cells (S2 Fig). These findings confirmed that this site in VP1 can indeed accommodate at least two Nb-encoding sequences, potentially allowing the insertion of heterologous Nb tandem repeats to target different receptors using the same AAV vector construct. In particular a combination of CD4-Nb1 and CD4-Nb3 might result in an even more CD4-specific AAV2 vector as it has been shown that both Nbs can bind CD4 in parallel, meaning their epitopes do not overlap [39].

The failure of CD4-Nb3-modified, as opposed to CD4-Nb1-modified VP2 capsid proteins to target the respective vector particles to CD4^+^ cells (Figs 3 and 4) may be explained by the obvious variability in the complementarity determining region 3 (CDR3) of these Nbs [39] (depicted in Fig 1A), which may negatively affect the overall structure of the VP2 protein, and thus the appropriate surface presentation of the CD4-Nb3.

Our AAV2 binding and internalization data suggests that VP1 CD4-Nb modified capsids do attach to both CD4 positive and negative cell membranes, whereas PepNb-modified capsids show a clearly reduced attachment to both cell lines (S1 Fig). This might be explained by weak and transient interactions of the CD4-Nbs with CD4-like epitopes present on both cell lines. Both CD4-Nbs bind to domain 1 of human CD4, resembling a common immunoglobulin variable domain (IgV) in the immunoglobulin superfamily [39]. Capsid internalization, however, is markedly increased for CD4-Nb1 on CD4 positive cells (HeLa TZMbl) and decreased in CD4 negative cells (HeLa wt), suggesting a CD4-specific internalization mechanism. The relatively low internalization rate of CD4-Nb3 capsids on CD4 positive cells might indicate a different uptake kinetic, as the affinity of CD4-Nb3 to CD4 is lower as for CD4-Nb1 (k_D_ 75nM and 5 nM, respectively) [39].

Clearly, the Nb-mediated capsid modifications presented here improved the ability of AAV2 vectors to target CD4^+^ cells, including primary human T lymphocytes. The latter are of particular interest for developing novel experimental therapies, for instance in the field of therapeutic genome editing [50], where direct, specific and efficient introduction of designer nucleases or recombinases [19, 51] into CD4^+^ lymphocytes is required. For example, the chromosomally integrated genome of human immunodeficiency virus (HIV; i.e. the provirus) persists indefinitely within CD4^+^ T cells and cannot be eradicated by classical antiretroviral therapy [20]. However, it has been demonstrated that *ex vivo* lentiviral vector-mediated introduction of HIV-specific designer recombinases into human T lymphocytes results in efficient provirus excision in HIV-infected humanized mice [18]. Thus, potential direct *in vivo* delivery of such an antiviral designer recombinase using CD4-Nb-targeted AAV gene vectors may ultimately enable the clinical development of a scalable curative therapy for HIV/AIDS.

In animal studies, systemic administration of AAV commonly requires high vector doses, for example ranging from ≥ 1x10^11^ to 1x10^13^ vector genomes per mouse or rhesus macaque [40, 48]. Generally, very high vector amounts are used to overcome vector off-target sequestration, but may also trigger undesired effects such as toxicity or immune responses [11, 52]. Thus, increasing vector specificity by capsid engineering may not only reduce the effective vector dose, but also minimize any pathogenic effects. First studies on the immunogenicity of Nbs used in clinical trials revealed a very low risk profile [53]. Furthermore, the CD4 Nbs used in this study were specifically and extensively characterized for their potential to stimulate human T cells and PMBCs, showing no increased activation compared to controls [39]. Hence, Nbs are unlikely to increase the risk of immune activation besides the AAV vectors alone.

In conclusion, our findings represent an important step towards the development of more potent AAV vectors for specific gene transfer into human CD4^+^ T lymphocytes.

## Supporting information

S1 Fig AAV2 vector binding and internalization analysis. AAV2 vector particle binding and internalization was assessed in HeLa wt and TZMbl cells by quantification of vector genome copies relative to cellular genomes. Particle binding was achieved by cultivating purified AAV2 vector preparations with target cells (10.000 gc/cell) at 4°C for 1h and subsequent stringent washing followed by cell harvest. For particle internalization, cells were incubated at 37°C for 1h after particle binding prior to washing and cell harvest (for details see Methods section). Data of three independent experiments, presented as fold to AAV2 opt particles with SD. Statistical significances are indicated with asterisks * p < .05, ** p < .01, *** p < .001, **** p < .0001 or specific p-values.
(TIF)

S2 Fig AAV2-VP1 bivalent-CD4 Nb specificity in mixed culture experiments. (A) Representative analysis of a mixed culture experiment comparing VP1-CD4-monovalent with -bivalent Nb constructs. HeLa wt (CD4 negative) were mixed with HeLa TZMbl (CD4 positive) in a ratio of 1:1 prior to AAV2 transduction and subsequently transduced with different virus dilutions. Three days post transduction cells were harvested, stained for CD4 and analyzed for eGFP expression by flow cytometry. (B) Summary of three independent HeLa mixed culture experiments. The relative frequencies of eGFP positive cells for CD4 positive and negative cells are plotted on the left. AAV2 CD4-specific transduction is calculated as a ratio from the individual cell populations (fold CD4 positive over CD4 negative). Fold changes are plotted on the right; n = 3, presented are means with SD, significant differences indicated with asterisks: * p < .05, ** p < .01, *** p < .001.
(TIF)

S1 Table Oligonucleotides used. List of all oligonucleotides used in this study.
(DOCX)

S2 Table Capsid and vector genome copy quantification. Capsid and vector genome copy quantification using ELISA and qPCR, respectively. Ratio between capsid and genomic titer is shown.
(DOCX)

S1 File Amino acid sequences of the final vector constructs. Amino acid sequences of all AAV2 VP constructs used in this study.
(PDF)

S1 Raw images (PDF)
