# Supplementary figures and images for: Improved targeting of human CD4+ T cells by nanobody-modified AAV2 gene therapy vectors
Source: PLoS One. 2021 Dec 20;16(12):e0261269. doi: 10.1371/journal.pone.0261269 (PMC8687595; doi:10.1371/journal.pone.0261269)

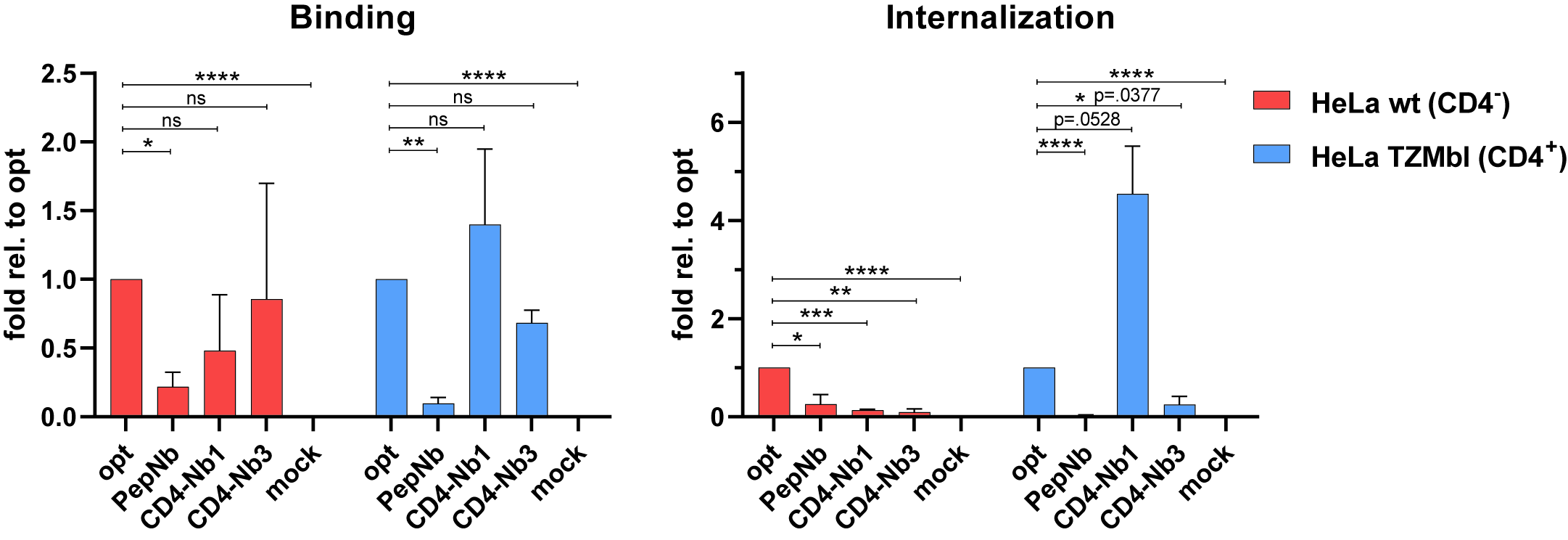

Supplement: S1 Fig — AAV2 vector particle binding and internalization was assessed in HeLa wt and TZMbl cells by quantification of vector genome copies relative to cellular genomes. Particle binding was achieved by cultivating purified AAV2 vector preparations with target cells (10.000 gc/cell) at 4°C for 1h and subsequent stringent washing followed by cell harvest. For particle internalization, cells were incubated at 37°C for 1h after particle binding prior to washing and cell harvest (for details see Methods section). Data of three independent experiments, presented as fold to AAV2 opt particles with SD. Statistical significances are indicated with asterisks * p < .05, ** p < .01, *** p < .001, **** p < .0001 or specific p-values. (TIF) [file pone.0261269.s001.tif]

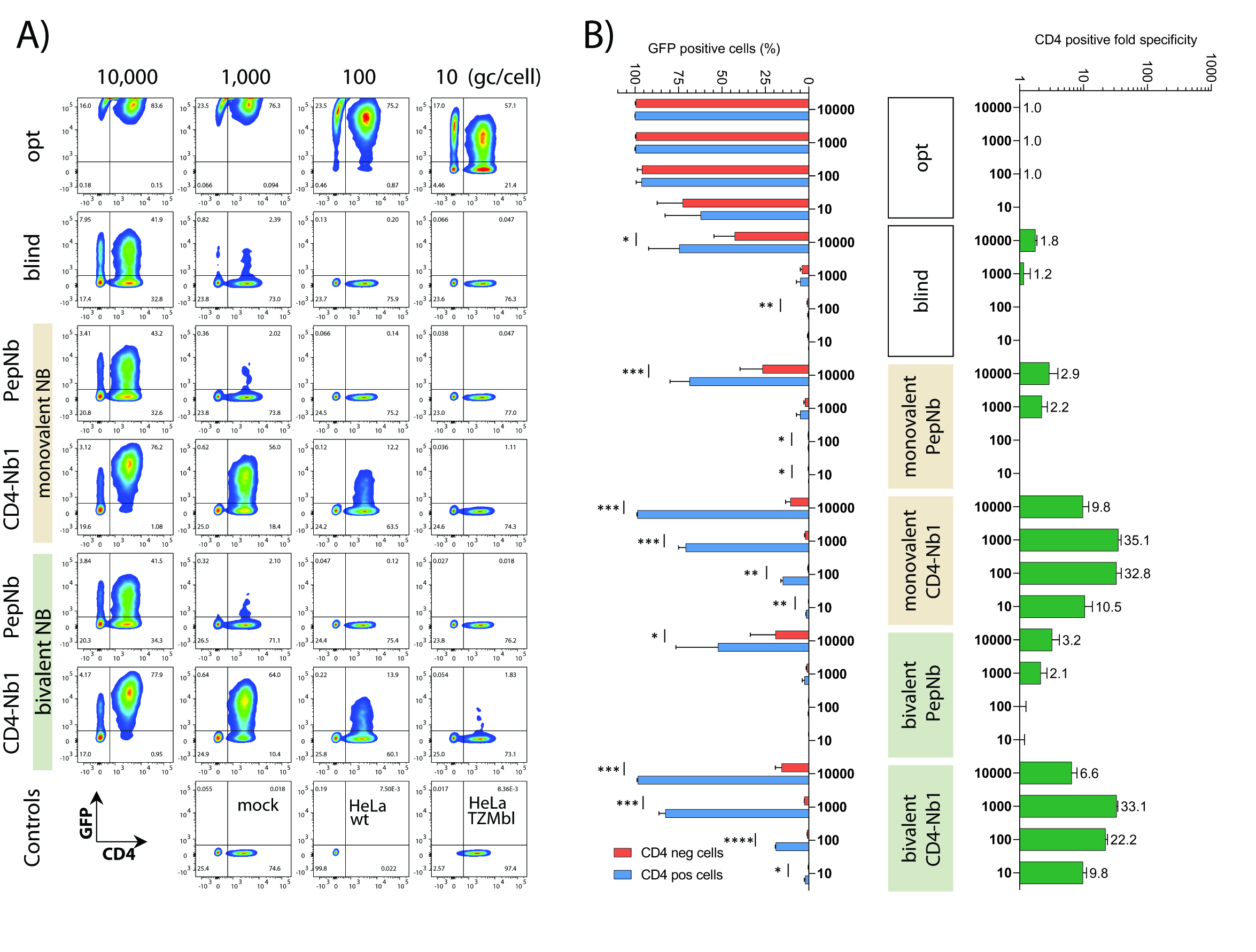

Supplement: S2 Fig — (A) Representative analysis of a mixed culture experiment comparing VP1-CD4-monovalent with -bivalent Nb constructs. HeLa wt (CD4 negative) were mixed with HeLa TZMbl (CD4 positive) in a ratio of 1:1 prior to AAV2 transduction and subsequently transduced with different virus dilutions. Three days post transduction cells were harvested, stained for CD4 and analyzed for eGFP expression by flow cytometry. (B) Summary of three independent HeLa mixed culture experiments. The relative frequencies of eGFP positive cells for CD4 positive and negative cells are plotted on the left. AAV2 CD4-specific transduction is calculated as a ratio from the individual cell populations (fold CD4 positive over CD4 negative). Fold changes are plotted on the right; n = 3, presented are means with SD, significant differences indicated with asterisks: * p < .05, ** p < .01, *** p < .001. (TIF) [file pone.0261269.s002.tif]
