# Supplementary material for: Improved targeting of human CD4+ T cells by nanobody-modified AAV2 gene therapy vectors
Source: PLoS One. 2021 Dec 20;16(12):e0261269. doi: 10.1371/journal.pone.0261269 (PMC8687595; doi:10.1371/journal.pone.0261269)
Supplement: S1 Table — List of all oligonucleotides used in this study. (DOCX) [file pone.0261269.s003.docx]

**S1 Table.**

| *Name* | *Sequence 5'-3'* | *Purpose* |
| --- | --- | --- |
| oMH16 | cctTCTAGAAtGGCTCCGGGAAAAAAG | ORF opt-blind VP2 |
| oMH18 | AACGGATCCTTACAGATTACGAGTCAG | ORF opt-blind VP2 |
| oMH19 | CAACgcACAAGCAGCTACCGCAGATG | SDM AAV2 cap blinding R585/588A |
| oMH20 | CCTgcCTGGAGGTTGGTAGATACAG | SDM AAV2 cap blinding R585/588A |
| oMH22 | AGTtCCTGTATTACTTGAGC | SDM AAV2 cap opt Y444F |
| oMH23 | GGTCGATGAGAGGATTCATG | SDM AAV2 cap opt Y444F |
| oMH24 | CAACAACAGTGAATtCTCGTGGACTG | SDM AAV2 cap opt T491V/Y500F |
| oMH25 | TTATCCGCAGATacCTTTGATACTCG | SDM AAV2 cap opt T491V/Y500F |
| oMH26 | GATtCCTGACTCGTAATCTG | SDM AAV2 cap opt Y730F |
| oMH27 | TGGTGCCAATGGGGCGAGGC | SDM AAV2 cap opt Y730F |
| oMH35 | GCGAAGCTTgccgccATGGCAGAGGTG | CD4-VP2-Nb4 PCR |
| oMH36 | TGCCTCGAGAGGAGACGGTGACCTGGG | CD4-VP2-Nb4 PCR |
| oMH37 | GCGAAGCTTgccgccATGGCAGAGGTG | CD4-VP2-Nb5 PCR |
| oMH38 | GGCCTCGAGaGGAAACGGTGACCGGAG | CD4-VP2-Nb5 PCR |
| oMH39 | GCGAAGCTTgccgccATGGCTGAGGTG | CD4-VP2-Nb1 PCR |
| oMH40 | GGCCTCGAGaTGAGACGGTGACCTGGG | CD4-VP2-Nb1 PCR |
| oMH41 | GCGAAGCTTgccgccATGGCCCACGTG | CD4-VP2-Nb3 PCR |
| oMH42 | GGCCTCGAGaGGAGACGGTGACCAGGG | CD4-VP2-Nb3 PCR |
| oMH43 | GCGAAGCTTgccgccATGGCCGAGGTG | CD4-VP2-Nb1a PCR |
| oMH44 | GGCCTCGAGAGGAGACGGTGACCTGG | CD4-VP2-Nb1a PCR |
| oMH87 | GCGAAGCTTgccgccatggctgacgtgcag | PepNb-VP2 PCR |
| oMH88 | GCCTCGAGAGGAGACGGTGACCtgggtg | PepNb-VP2 PCR |
| oMH45 | TCAGGTAaGGCTGCCGATGG | SDM VP1 start codon inactivation (sc) |
| oMH46 | TTTAAATCATTTATTGTTCAAAGATGC | SDM VP1 start codon inactivation (sc) |
| oMH47 | TCTTCCttATTGGCTCGAGG | SDM VP2/3 splice acceptor inactivation (SA) |
| oMH48 | TAACCATCGGCAGCCATACC | SDM VP2/3 splice acceptor inactivation (SA) |
| oMH67 | ACTTGGAGTGTTTGTTCTGCTCAAGTAATACAGG | SDM NotI site implementation |
| oMH68 | GCCGCggcggcggcggcgcgCTTCAGTTTTCTCAG | SDM NotI site implementation |
| oMH69 | ggcggcggcggcagcggcggcggcggcagcggcggcggcggcagcggcggcggcggcagcggcggcggcggcagcGCG | oligo annealing 5' linker block |
| oMH70 | CGCgctgccgccgccgccgctgccgccgccgccgctgccgccgccgccgctgccgccgccgccgctgccgccgccgcc | oligo annealing 5' linker block |
| oMH075 | gcggcagcggcggcggcggcagcGCtATGGCTGAGGTGCAG | CD4-VP1-Nb1 PCR |
| oMH076 | AACTGAAGcgcgccgccgccgccGCtTGAGACGGTGACCTG | CD4-VP1-Nb1 PCR |
| oMH077 | gcggcagcggcggcggcggcagcGCtATGGCCGAGGTGCAG | CD4-VP1-Nb1a PCR |
| oMH078 | AACTGAAGcgcgccgccgccgccGCtGGAGACGGTGACCTG | CD4-VP1-Nb1a PCR |
| oMH079 | gcggcagcggcggcggcggcagcGCtATGGCAGAGGTGCAG | CD4-VP1-Nb4 PCR |
| oMH080 | AACTGAAGcgcgccgccgccgccGCtGGAGACGGTGACCTG | CD4-VP1-Nb4 PCR |
| oMH083 | gcggcagcggcggcggcggcagcGCtATGGCCCACGTGCAG | CD4-VP1-Nb3 PCR |
| oMH084 | AACTGAAGcgcgccgccgccgccGCtGGAGACGGTGACCAG | CD4-VP1-Nb3 PCR |
| oMH095 | gcggcagcggcggcggcggcagcGCtatggctgacgtgcag | PepNb-VP1 PCR |
| oMH096 | AACTGAAGcgcgccgccgccgccGCtGGAGACGGTGAC | PepNb-VP1 PCR |
| oMH085 | GCggcggcggcggcgcgCTTCAGTTTTC | overlap extension PCR cap gene |
| oMH086 | AGGTACACATCTCTGTCCTGCCAGACCA | overlap extension PCR cap gene |
| oMH093 | GCGATCTCGATGCTTTATTT | AAV qPCR SV40pA |
| oMH094 | AGAGGGAGTGGACTAGTTT | AAV qPCR SV40pA |
| HBG-F | CTT AAT GCC TTA ACA TTG TGT ATA A | Genomic qPCR HBG |
| HBG-R | GAA TAT GCA AAT AAG CAC ACA TAT AT | Genomic qPCR HBG |
| HBG-probe | (FAM)-ACT TTA CAC AGT CTG CCT AGT ACA TTA C-(TAMRA) | Genomic qPCR HBG |
