# Supplementary material for: Improved targeting of human CD4+ T cells by nanobody-modified AAV2 gene therapy vectors
Source: PLoS One. 2021 Dec 20;16(12):e0261269. doi: 10.1371/journal.pone.0261269 (PMC8687595; doi:10.1371/journal.pone.0261269)
Supplement: S2 Table — Capsid and vector genome copy quantification using ELISA and qPCR, respectively. Ratio between capsid and genomic titer is shown. (DOCX) [file pone.0261269.s004.docx]

| **AAV2 capsid variant** | **Genomic titer [gc/µL]** | **Capsid titer [cap/µL]** | **Ratio cap/gc** |
| --- | --- | --- | --- |
| **opt** | 1.41x10^8^ | 1.57x10^9^ | 11.13 |
| **blind** | 1.18x10^8^ | 8.15x10^8^ | 6.91 |
| **VP1-CD4-Nb1** | 5.47x10^7^ | 3.92x10^8^ | 7.17 |
| **VP2-CD4-Nb1** | 1.32x10^8^ | 1.04x10^9^ | 7.88 |
| **VP1-biCD4-Nb1** | 5.78x10^7^ | 5.05x10^8^ | 8.74 |

**S2 Table.**
