# Supplementary material for: Improved targeting of human CD4+ T cells by nanobody-modified AAV2 gene therapy vectors
Source: PLoS One. 2021 Dec 20;16(12):e0261269. doi: 10.1371/journal.pone.0261269 (PMC8687595; doi:10.1371/journal.pone.0261269)
Supplement: S1 File — Amino acid sequences of all AAV2 VP constructs used in this study. (PDF) [file pone.0261269.s005.pdf]

S1 File.

Legend:
AAV2 original sequence
Modified amino acid
Amino acid change to disrupt HSPG binding
Sequence replaced by nanobody insertion
Nanobody sequence
N-terminal/central linker sequence
C-terminal linker sequence

AAV2 VP1 Protein (blind and optimized)
MAADGYLPDWLEDTLSEGIRQWWKLKPGPPPPKPAERHKDDSRGLVLPGYKYLGPFNGLDKGEPVNEADAAALE
HDKAYDRQLDSGDNPYLKYNHADAEFQERLKEDTSFGGNLGRAVFQAKKRVLEPLGLVEEPVKTAPGKKRPVEHSP
VEPDSSSGTGKAGQQPARKRLNFGQTGDADSVPDPQPLGQPPAAPSGLGTNTMATGSGAPMADNNEGADGVG
NSSGNWHCDSTWMGDRVITTSTRTWALPTYNNHLYKQISSQSGASNDNHYFGYSTPWGYFDFNRFHCHFSPRD
WQRLINNNWGFRPKRLNFKLFNIQVKEVTQNDGTTTIANNLTSTVQVFTDSEYQLPYVLGSAHQGCLPPFPADVF
MVPQYGYLTLNNGSQAVGRSSFYCLEYFPSQMLRTGNNFTFSYTFEDVPFHSSYAHSQSLDRLMNPLIDQFLYYLSR
TNTPS***GTTTQSR***LQFSQAGASDIRDQSRNWLPGPCYRQQRVSKVSADNNNSEFSWTGATKYHLNGRDSLVNPGP
AMASHKDDEEKFFPQSGVLIFGKQGSEKTNVDIEKVMITDEEEIRTTNPVATEQYGSVSTNLQAGNAQAATADVNT
QGVLPGMVWQDRDVYLQGPIWAKIPHTDGHFHPSPLMGGFGLKHPPPQILIKNTPVPANPSTTFSAAKFASFITQ
YSTGQVSVEIEWELQKENSKRWNPEIQYTSNYNKSVNVDFTVDTNGVYSEPRPIGTRFLTRNL*

VP1-PepNb

GGGGSGGGGSGGGGSGGGGSGGGGGSAMADVQLQESGGGLVQPGGSLRLSCAASGNIVSIDAAGWFRQAPG
KQREPVATILTGGATNYADSVKGRFTISRDNAKNTVYLQMNSLKPEDTAVYYCYAPMIYYGGRYSDYWGQGTQVT
VSSGGGGA

VP1-CD4-Nb4

GGGGSGGGGSGGGGSGGGGSGGGGSAMAEVQLQESGGALAQPGGSLRLSCAVSGFTFGTYGMGWLRQAPGK
GREFVAAINWIHGGEIYADSVKGRFTISKDNAKNTLYLQMDSLKPEDTAVYYCAARSGTSLYTGVDEYQYWGQGT
QVTVSSGGGGA

VP1-CD4-Nb5 – not applicable

VP1-CD4-Nb1

GGGGSGGGGSGGGGSGGGGSGGGGSAMAEVQLVESGGGLVQPGGSLRLSCAASGFTFSKLAMSWHREPPGKG
REWLADIDSSGDTTDYLASVKGRFTISRDNAKNTLYLQMDSLKSEDTGVYYCASREDPPGYWGQGTQVTVSSGGG
GA

VP1-CD4-Nb3

GGGGSGGGGSGGGGSGGGGSGGGGSAMAHVQLVESGGGLVQPGGSLRLSCAVSGFALEYYAIGWFRQAPGKE
RERVACMSASGGVINYSESVKGRFTISRDNAKNTVYLQMNTLKPEDTAVYYCAAEKAYYGSSWAECYLMMDYWG
KGTLVTVSSGGGGA

VP1-CD4-Nb1a

GGGGSGGGGSGGGGSGGGGSGGGGGSGGGGSAMAEVQLVESGGGLVQPGGSLRLSCVASGFTFSSSGMTWV
RQAPGKGLEWVSDINSGGDTTDYLASVKGRFTISRDNAKNTLYLQMDSLKSEDTGVYYCASREDPPGYWGQGIQV
TVSSGGGGA

VP1-biCD4-Nb1

GGGGSGGGGSGGGGSGGGGSGGGGSAMAEVQLVESGGGLVQPGGSLRLSCAASGFTFSKLAMSWHREPPGKG
REWLADIDSSGDTTDYLASVKGRFTISRDNAKNTLYLQMDSLKSEDTGVYYCASREDPPGYWGQGTQVTVSASGG
GGSGGGGSGGGGSGGGGSGGGGSMAEVQLVESGGGLVQPGGSLRLSCAASGFTFSKLAMSWHREPPGKGREW
LADIDSSGDTTDYLASVKGRFTISRDNAKNTLYLQMDSLKSEDTGVYYCASREDPPGYWGQGTQVTVSSGGGGA

VP1-biPepNB

GGGGSGGGGSGGGGSGGGGSGGGGSAMADVQLQESGGGLVQPGGSLRLSCAASGNIVSIDAAGWFRQAPGK
QREPVATILTGGATNYADSVKGRFTISRDNAKNTVYLQMNSLKPEDTAVYYCYAPMIYYGGRYSDYWGQGTQVTV
SSASGGGGSGGGGSGGGGSGGGGSGGGGSMADVQLQESGGGLVQPGGSLRLSCAASGNIVSIDAAGWFRQAP
GKQREPVATILTGGATNYADSVKGRFTISRDNAKNTVYLQMNSLKPEDTAVYYCYAPMIYYGGRYSDYWGQGTQV
TVSSGGGGA

AAV2 VP2 Protein (blind and optimized)
MAPGKKRPVEHSPVEPDSSSGTGKAGQQPARKRLNFGQTGDADSVPDPQPLGQPPAAPSGLGTNTMATGSGAP
MADNNEGADGVGNSSGNWHCDSTWMGDRVITTSTRTWALPTYNNHLYKQISSQSGASNDNHYFGYSTPWGYF
DFNRFHCHFSPRDWQRLINNNWGFRPKRLNFKLFNIQVKEVTQNDGTTTIANNLTSTVQVFTDSEYQLPYVLGSAH
QGCLPPFPADVFMVPQYGYLTLNNGSQAVGRSSFYCLEYFPSQMLRTGNNFTFSYTFEDVPFHSSYAHSQSLDRLM
NPLIDQFLYYLSRTNTPSGTTTQSRLQFSQAGASDIRDQSRNWLPGPCYRQQRVSKVSADNNNSEFSWTGATKYHL
NGRDSLVNPGPAMASHKDDEEKFFPQSGVLIFGKQGSEKTNVDIEKVMITDEEEIRTTNPVATEQYGSVSTNLQAG
NAQAATADVNTQGVLPGMVWQDRDVYLQGPIWAKIPHTDGHFHPSPLMGGFGLKHPPPQILIKNTPVPANPST
TFSAAKFASFITQYSTGQVSVEIEWELQKENSKRWNPEIQYTSNYNKSVNVDFTVDTNGVYSEPRPIGTRFLTRNL*

VP2-PepNb

MADVQLQESGGGLVQPGGSLRLSCAASGNIVSIDAAGWFRQAPGKQREPVATILTGGATNYADSVKGRFTISRDN
AKNTVYLQMNSLKPEDTAVYYCYAPMIYYGGRYSDYWGQGTQVTVSSRGGSGGSGGSGGSGGSR-VP2

VP2-CD4-Nb4

MAEVQLQESGGALAQPGGSLRLSCAVSGFTFGTYGMGWLRQAPGKGREFVAAINWIHGGEIYADSVKGRFTISKD
NAKNTLYLQMDSLKPEDTAVYYCAARSGTSLYTGVDEYQYWGQGTQVTVSSRGGSGGSGGSGGSR-VP2

VP2-CD4-Nb5

MAEVQLQESGGGLVQAGGSLRLSCKVSGRTFGTYAMGWFRQAPGKEREFVAVISWIKGNTYVADSVLGRFTISRD
NARNWLHLQMYSLKPEDTATYYCAASSTVTLPTTLDPYTYWGQGTPVTVSSRGGSGGSGGSGGSR-VP2

VP2-CD4-Nb1

MAEVQLVESGGGLVQPGGSLRLSCAASGFTFSKLAMSWHREPPGKGREWLADIDSSGDTTDYLASVKGRFTISRD
NAKNTLYLQMDSLKSEDTGVYYCASREDPPGYWGQGTQVTVSSRGGSGGSGGSGGSR-VP2

VP2-CD4-Nb3

MAHVQLVESGGGLVQPGGSLRLSCAVSGFALEYYAIGWFRQAPGKERERVACMSASGGVINYSESVKGRFTISRD
NAKNTVYLQMNTLKPEDTAVYYCAAEKAYYGSSWAECYLMMDYWGKGTLVTVSSRGGSGGSGGSGGSR-VP2

VP2-CD4-Nb1a

MAEVQLVESGGGLVQPGGSLRLSCVASGFTFSSSGMTWVRQAPGKGLEWVSDINSGGDTTDYLASVKGRFTISRD
NAKNTLYLQMDSLKSEDTGVYYCASREDPPGYWGQGIQVTVSSRGGSGGSGGSGGSR-VP2
