# Supplementary material for: Improved targeting of human CD4+ T cells by nanobody-modified AAV2 gene therapy vectors
Source: PLoS One. 2021 Dec 20;16(12):e0261269. doi: 10.1371/journal.pone.0261269 (PMC8687595; doi:10.1371/journal.pone.0261269)

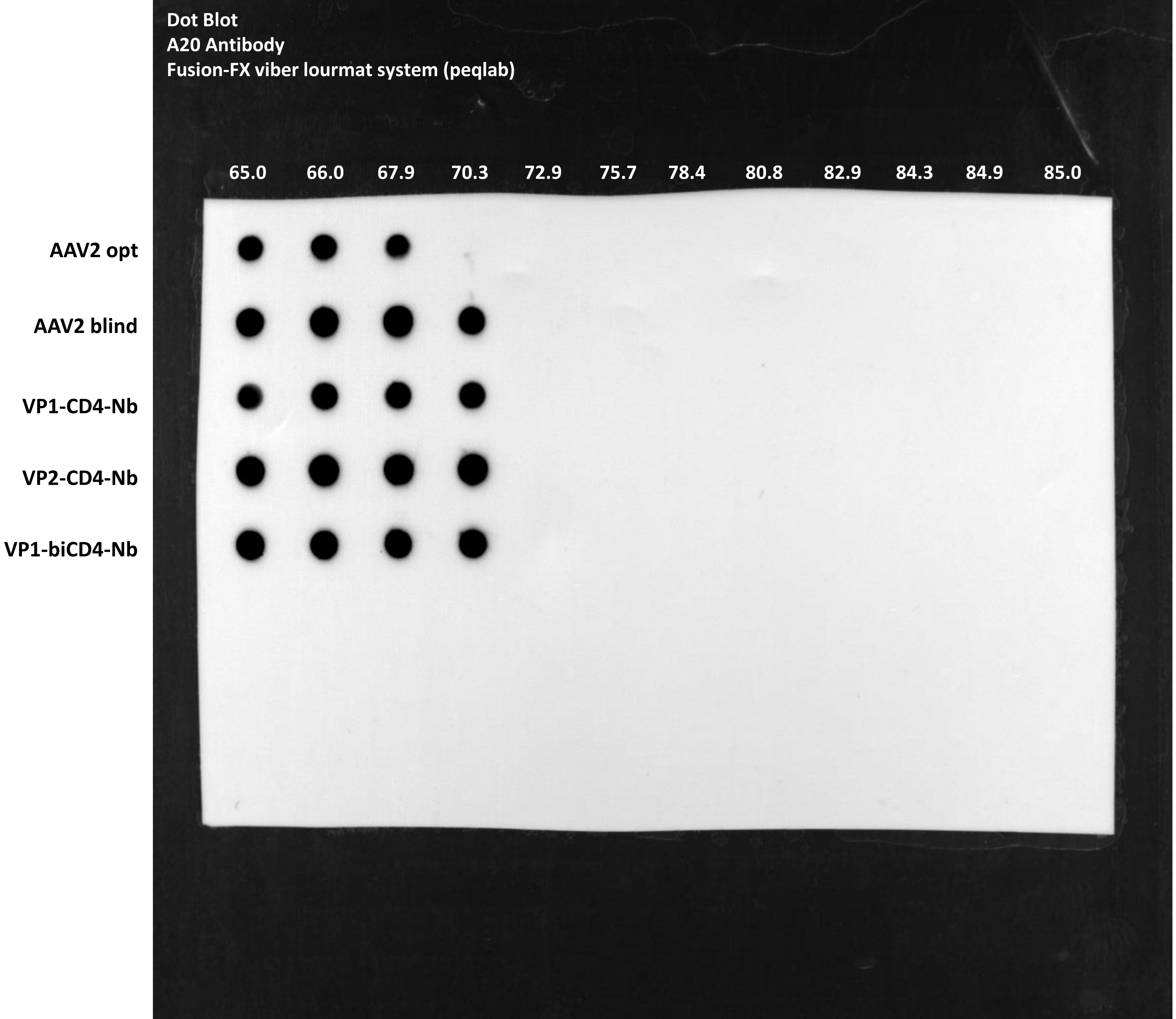
Dot Blot
A20 Antibody
Fusion-FX viber lourmat system (peqlab)
65.0
66.0
67.9
70.3
72.9
75.7
78.4
80.8
82.9
84.3
84.9
85.0
AAV2 opt
AAV2 blind
VP1-CD4-Nb
VP2-CD4-Nb
VP1-biCD4-Nb

Western Blot
A69 Antibody
Fusion-FX viber lourmat system (peqlab)

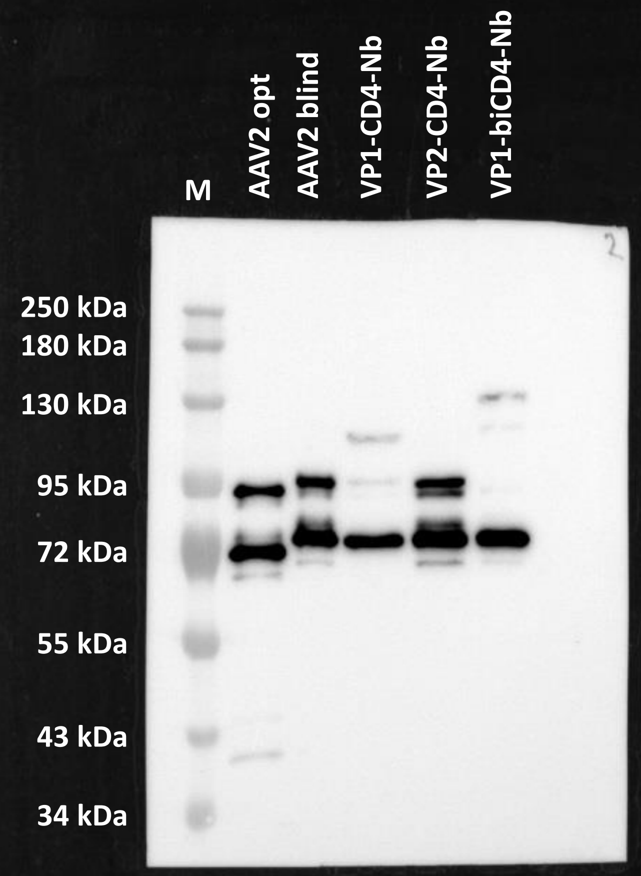

Supplement: S1 Raw images — (PDF) [file pone.0261269.s006.pdf]
